# Outcomes of natalizumab treatment within 3 years of relapsing-remitting multiple sclerosis diagnosis: a prespecified 2-year interim analysis of STRIVE

**DOI:** 10.1186/s12883-019-1337-z

**Published:** 2019-06-08

**Authors:** Jai Perumal, Robert J. Fox, Roumen Balabanov, Laura J. Balcer, Steven Galetta, Shavy Makh, Sourav Santra, Christophe Hotermans, Lily Lee

**Affiliations:** 1 Weill Cornell Multiple Sclerosis Center, New York, NY USA; 2 Mellen Center for Multiple Sclerosis, Cleveland Clinic, Cleveland, OH USA; 3 Northwestern University, Chicago, IL USA; 4 New York University School of Medicine, New York, NY USA; 5 Biogen, 225 Binney St, Cambridge, MA 02142 USA

**Keywords:** Relapsing-remitting multiple sclerosis, Natalizumab, Anti-JCV antibody, No evidence of disease activity, Brain atrophy, Optical coherence tomography, Patient-reported outcomes

## Abstract

**Background:**

STRIVE is a multicenter, observational, open-label, single-arm study of natalizumab in anti–JC virus (JCV) seronegative patients with early relapsing-remitting multiple sclerosis (RRMS). The objective of this prespecified 2-year interim analysis was to determine the effectiveness of natalizumab in establishing and maintaining no evidence of disease activity (NEDA) in early RRMS.

**Methods:**

Patients aged 18–65 years had an RRMS diagnosis < 3 years prior to screening, an Expanded Disability Status Scale (EDSS) score ≤ 4.0, and anti-JCV antibody negative status. Magnetic resonance imaging was performed at baseline and yearly thereafter. Cumulative probabilities of 24-week–confirmed EDSS worsening and improvement were evaluated at 2 years. NEDA (no 24-week–confirmed EDSS worsening, no relapses, no gadolinium-enhancing lesions, and no new/newly enlarging T2-hyperintense lesions) was evaluated over 2 years. The Symbol Digit Modalities Test (SDMT) and Multiple Sclerosis Impact Score (MSIS-29) were assessed at baseline and 1 and 2 years. Statistical analysis used summary statistics and frequency distributions.

**Results:**

The study population (*N* = 222) had early RRMS, with mean (standard deviation [SD]) time since diagnosis of 1.6 (0.77) years and mean (SD) baseline EDSS score of 2.0 (1.13). NEDA was achieved in 105 of 187 patients (56.1%) during year 1 and 120 of 163 (73.6%) during year 2. Over 2 years, 76 of 171 patients (44.4%) attained overall NEDA. Probabilities of 24-week–confirmed EDSS worsening and improvement were 14.1% and 28.4%, respectively. After 2 years, patients exhibited significant improvements from baseline in SDMT (*n* = 158; mean [SD]: 4.3 [11.8]; *p* < 0.001) and MSIS-29 physical (*n* = 153; mean [SD]: − 3.9 [14.7]; *p* = 0.001), psychological (*n* = 152; mean [SD]: − 2.0 [7.9]; *p* < 0.001), and quality-of-life (*n* = 153; mean [SD]: − 6.0 [21.3]; *p* < 0.001) scores.

**Conclusions:**

These results support natalizumab’s effectiveness over 2 years, during which nearly half of early RRMS patients achieved NEDA. During year 2, nearly 75% of patients exhibited NEDA. Over 2 years, patients continued to experience significant cognitive and quality-of-life benefits. These results are limited by the lack of a comparator group to determine the extent of a placebo effect.

**Trial registration:**

clinicaltrials.gov, NCT01485003, registered 5 December 2011.

**Electronic supplementary material:**

The online version of this article (10.1186/s12883-019-1337-z) contains supplementary material, which is available to authorized users.

## Background

In relapsing-remitting multiple sclerosis (RRMS) patients, natalizumab significantly reduced disease activity as reflected by brain magnetic resonance imaging (MRI), clinical relapse rate, and confirmed disability worsening compared with placebo over 2 years in the AFFIRM trial [1]. In a post hoc analysis of AFFIRM, natalizumab-treated patients, especially those with high disease activity, were significantly more likely than placebo-treated patients to have no evidence of disease activity (NEDA) [2].

Natalizumab treatment earlier in the RRMS disease course may be associated with better clinical outcomes [3, 4]. Clinical outcome measures (e.g., relapse rate and Expanded Disability Status Scale [EDSS] score) and patient-reported outcome (PRO) measures (e.g., quality-of-life assessments) provide valuable information, enabling healthcare providers to evaluate treatment effects and disease control in multiple sclerosis (MS) [5–7]. Even in the early stages of RRMS, patients have reported reductions in their quality of life and ability to work [8], suggesting a need for early and effective treatment. In addition, cognition, as assessed by performance on the Symbol Digit Modalities Test (SDMT), has been shown to predict health-related quality of life (HRQoL) [9], highlighting the importance of assessing cognitive function during comprehensive MS patient care.

Natalizumab treatment is associated with a risk of progressive multifocal leukoencephalopathy (PML), a central nervous system infection caused by the JC virus (JCV) [10]. Longer treatment duration (especially > 2 years), prior immunosuppressant use, and the presence of anti-JCV antibodies in serum are associated with greater risk of developing natalizumab-associated PML [10, 11]. The benefit/risk profile of natalizumab is enhanced when natalizumab is used to treat patients at very low risk of developing PML; this study includes only anti-JCV antibody negative patients, whose PML risk is estimated to be < 0.1 in 1000 [10, 11].

This 2-year analysis of the Observational Study of Tysabri in Early Relapsing-Remitting MS in Anti-JCV Antibody Negative Patients (STRIVE) assessed the effectiveness of natalizumab at establishing and maintaining NEDA, defined as no 24-week–confirmed disability worsening, no relapses, no gadolinium-enhancing (Gd+) lesions, and no new or newly enlarging T2-hyperintense lesions, in patients with early RRMS (i.e., < 3 years from diagnosis). This analysis also evaluated changes in cognition, capacity for work, quality of life, visual acuity, and retinal nerve fiber layer (RNFL) thickness in an early RRMS population. Preliminary results from this study have been presented previously [12–14].

## Methods

### Study design

STRIVE (clinicaltrials.gov NCT01485003) is an ongoing, 4-year, prospective, open-label, multicenter, single-country phase 4 study of anti-JCV antibody negative patients with early RRMS initiating natalizumab treatment. The study started on February 7, 2012. In this prespecified 2-year interim analysis of STRIVE, patients from 47 study sites in the United States received 300 mg intravenous natalizumab every 4 weeks according to the US natalizumab product label [15].

The study included patients aged 18–65 years who had an RRMS diagnosis within 3 years prior to screening, an EDSS score ≤ 4.0, and a negative test for anti-JCV antibodies within 6 months prior to screening. Patients were either treatment naive with respect to disease-modifying therapies (DMTs) or had received prior DMTs other than natalizumab for ≤ 36 months. The study excluded patients who had prior natalizumab treatment, were anti-JCV antibody positive at any point prior to screening, or had a prior history of immunosuppressant use (e.g., mitoxantrone, azathioprine, methotrexate, cyclophosphamide, mycophenolate, cladribine, or rituximab).

Prior to enrollment, all patients provided written informed consent, and approval was granted by an independent ethics committee for each study site (full list available in Additional file 1). The study was performed in accordance with Good Clinical Practice Guidelines.

### Assessments

The primary endpoint in this analysis was the proportion of patients who achieved overall NEDA at 2 years. Overall NEDA included attainment of both clinical NEDA, defined as no relapses and no EDSS worsening sustained for 24 weeks, and MRI NEDA, defined as no Gd + lesions and no new or newly enlarging T2-hyperintense lesions. EDSS worsening was defined as a ≥ 0.5-point increase from a baseline EDSS score of ≥ 6.0, a ≥ 1.0-point increase from a baseline EDSS score of 1.0 to 6.0, or a ≥ 1.5-point increase from a baseline score of 0.0. For the yearly evaluation of NEDA, only patients with all required assessments were included. For the evaluation of NEDA over 2 years, patients who did not achieve NEDA in year 1 and who had ≥1 missing assessment at year 2 were included in the analysis population.

A number of key secondary endpoints were assessed at 2 years, including the proportions of patients who achieved clinical NEDA and MRI NEDA. Baseline characteristics that predicted overall NEDA (e.g., age [< 40 vs. ≥ 40 years], the number of relapses prior to natalizumab infusion [≤ 1 vs. > 1], EDSS score [≤2.0 vs. > 2.0], time from MS diagnosis [≤ 2.0 vs. > 2.0 years], and T2 lesion volume [≤ 4 mL vs. > 4 mL]) were identified. Other secondary endpoints included annualized relapse rate (ARR), time to first relapse, 24-week–confirmed disability worsening, and 24-week–confirmed disability improvement (defined as a ≥ 1.0-point decrease from baseline EDSS score of ≥ 2.0). Improvement was not assessed in patients with baseline EDSS scores < 2.0.

Relapse assessments were ongoing throughout the first 2 years of the study, and all relapses that started within 2 years of the first natalizumab dose were included in this analysis. A clinical relapse was defined as new or recurrent neurological symptoms not associated with fever, lasting at least 24 h, and followed by a period of 30 days of stability or improvement. New or recurrent neurological symptoms that occurred less than 30 days after the onset of a protocol-defined relapse were considered part of the same relapse. EDSS score was assessed every 6 months. Since MRI data were collected annually in STRIVE, secondary endpoints assessed by MRI were evaluated at the 2-year visit. NeuroRx Research (Montreal, Quebec, Canada) read and interpreted all MRIs.

Additional secondary endpoints evaluated changes in cognitive function and quality of life. Evaluation of cognitive function was performed using the SDMT and assessed at baseline and after 1 and 2 years of natalizumab treatment. The SDMT measures cognitive function using a timed test involving matching of geometric designs and verbal numerical responses [16, 17], with a higher score (a higher number of correct matches within a given time frame) indicating better cognitive function. Quality of life was measured by the Multiple Sclerosis Impact Scale (MSIS-29), an HRQoL measure that assesses a patient’s perception of the impact of MS on his or her day-to-day life, with a higher score (range, 0–100) indicating worse HRQoL [18]. Capacity for work was measured by the Work Productivity and Activity Impairment Questionnaire (WPAI), which quantitatively assesses absenteeism, presenteeism (i.e., going to work despite an illness that hinders functioning), work productivity loss, and daily activity impairment [19], with a lower score indicating higher work capacity. MSIS-29 and WPAI outcomes were evaluated at baseline and after 1 and 2 years of treatment.

Secondary endpoints related to RNFL thickness (measured by optical coherence tomography [OCT]) and visual acuity (VA) were assessed in a subset of patients (at centers with OCT capabilities) at baseline and 2 years. High-contrast VA was measured using the Snellen Eye Chart, and low-contrast VA was measured using the low-contrast Sloan letter charts (Precision Vision, LaSalle, IL, USA) at 2.5 and 1.25% levels. Patients were asked to read each of the two charts at a 2-m distance. Previously established definitions of VA worsening [20] and improvement [21] were used.

For all patients, serum and plasma were tested for the presence of anti-JCV antibodies using an enzyme-linked immunosorbent assay (ELISA) at screening and every 6 months thereafter. Once enrolled, patients testing positive for anti-JCV antibodies during the study were allowed to continue with natalizumab treatment at the discretion of the treating neurologist. Patients were also encouraged to continue with study follow-up through 4 years and the end of the study regardless of their anti-JCV antibody status or natalizumab treatment decision. Safety endpoints were assessed as incidence of serious adverse events (SAEs). SAE assessments were ongoing throughout the study, with each SAE determined to be either related or not related to study treatment.

All assessments were performed at the specified times ±1 month.

### Statistical analyses

The prespecified 2-year analyses included data up to 2 years for all patients, regardless of whether they were still enrolled in the study after 2 years or had discontinued the study by that point. The intent-to-treat (ITT) population was defined as all enrolled patients who completed informed consent and received at least one natalizumab dose. The study was planned to enroll 300 patients based on feasibility. This study sample size was not based on test statistics for comparison between two groups. With a sample size of 300 patients and an assumed proportion of disease free patients of (40%, based on results from the AFFIRM trial in which 37% of patients were free of disease activity over 2 years^2^), the two-sided 95% confidence interval (CI) using large sample normal approximation allowed an extension of ±6% from the observed proportion (ie, a 95% CI of 34–46%). In general, continuous variables were analyzed using summary statistics and categorical variables were analyzed using frequency distributions. Analyses were conducted using two-sided tests with a type I error rate of 0.05.

The NEDA analyses used observed data only. Patients missing measurements who achieved NEDA on all available measurements were excluded, whereas patients missing measurements who had evidence of disease activity on ≥ 1 measurement were considered as not achieving NEDA. In the assessment of overall NEDA, clinical NEDA, and MRI NEDA at 12 months differentiated by use of any prior DMT treatment at baseline, the corresponding *p*-value was based on a chi-square test. Logistic regression was utilized to assess the association between NEDA (overall, clinical, or MRI) and patient baseline characteristics. A paired *t*-test was used to assess within-subject change from baseline for continuous response variables.

The ARR at each time point was analyzed using a negative binomial regression model, adjusting for potential confounding factors. The corresponding *p*-value was based on a repeated negative binominal model for estimated pre−/post-natalizumab infusion annualized relapse. A Kaplan-Meier analysis and Cox proportional-hazards modeling were used to evaluate time to relapse, time to 24-week–confirmed disability worsening, and time to 24-week–confirmed disability improvement. PRO changes from baseline were assessed using a paired *t*-test. Generalized estimating equation models, adjusted for age and baseline RNFL thickness, were used to assess least-squares mean changes from baseline in RNFL thickness (left and right eye). For SDMT, WPAI, and MSIS-29, *p*-values and 95% CIs for change from baseline were based on a paired *t*-test. In the ITT population, the total number and incidence of SAEs were estimated by the proportion of patients experiencing ≥ 1 SAE.

## Results

### Patients

**Fig. 1:**
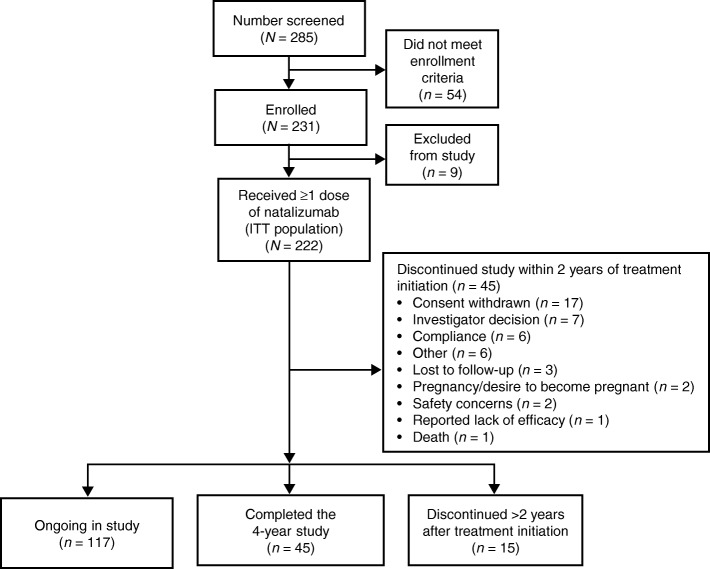
Patient disposition up to 2 years. ITT = intent to treat

STRIVE enrolled 231 patients (Fig. 1). Among the 229 patients with follow-up data, mean (SD) follow-up for this 2-year analysis was 23.7 (5.7) months. At baseline, the ITT population (*N* = 222) had a mean time from MS symptom onset of 3.0 years and a mean time from MS diagnosis of 1.6 years (Table 1). Patients in the ITT population had a mean of 1.4 relapses in the year prior to enrollment and a mean EDSS score of 2.0. Half of the patients (111 of 222) had not received DMTs prior to enrollment (Table 1).

**Table 1:** Patient characteristics at baseline (ITT population)

Baseline characteristic	Natalizumab-treated patients (*N* = 222)
Age, years	
Mean (SD)	34.0 (8.97)
Median (min, max)	32 (18, 64)
Female, *n* (%)	161 (72.5)
Time from MS symptom onset, mean (SD), years	3.0 (2.59)
Time from diagnosis of MS, mean (SD), years	1.6 (0.77)
Prior DMT treatments, *n* (%)	111 (50.0)
Number of relapses in the past 12 months, mean (SD)	1.4 (1.15)
EDSS score	
Mean (SD)	2.0 (1.13)
Median (min, max)	2.0 (0.0, 6.5)
T1 lesion volume, median (min, max), mL	0.6 (0, 23.8)^a^
T2 lesion volume, median (min, max), mL	3.9 (0, 61.6)^a^
Gd + lesions, mean (SD)	2.5 (7.52)^a^
Patients with no Gd + lesions, *n* (%)	114 (57.9)^a^
SDMT score, mean (SD)	52.1 (14.02)^b^
MSIS-29 score, mean (SD)	
Physical	42.2 (19.05)^c^
Psychological	21.9 (9.29)^c^
Quality of life	64.2 (27.07)^c^

*DMT* disease-modifying therapy, *EDSS* Expanded Disability Status Scale, *Gd +* gadolinium-enhancing, *ITT* intent to treat, *MS* multiple sclerosis, *SD* standard deviation, *SDMT* Symbol Digit Modalities Test

^a^*n* = 197

^b^*n* = 221

^c^*n* = 217

Of the 222 patients in the ITT population, 196 patients tested negative for anti-JCV antibodies at screening; an additional 26 patients had a negative anti-JCV antibody test within 6 months prior to screening. At 1 and 2 years, anti-JCV antibody test results were available for 178 and 151 patients, respectively, 164 (92.1%) and 133 (88.1%) of whom were anti-JCV antibody negative. The decrease in anti-JCV testing results is accounted for, in part, by the discontinuation of 45 patients (20.3%) in the ITT population within the first 2 years. Reasons for discontinuation included withdrawal of consent (7.7%), investigator decision (2.7%), compliance (2.7%), and patient lost to follow-up (1.4%). Safety concerns, pregnancy/desire to become pregnant, or lack of efficacy were cited by less than 1% of participants.

### NEDA

**Fig. 2:**
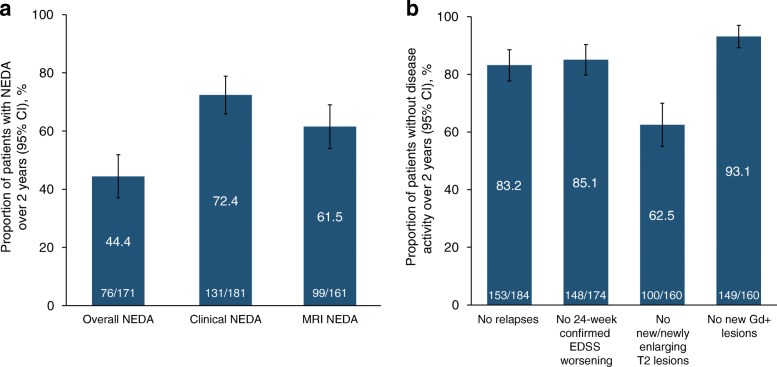
Proportions of patients with (**a**) overall, clinical, and MRI NEDA maintained over 2 years, and (**b**) no relapses, no confirmed disability worsening, and no MRI lesions over 2 years. Patients missing measurements who achieved NEDA on all available measurements were excluded, whereas patients missing measurements who had evidence of disease activity on ≥1 measurement were considered as not achieving NEDA. CI = confidence interval; EDSS = Expanded Disability Status Scale; MRI = magnetic resonance imaging; NEDA = no evidence of disease activity

Among the 171 patients assessed, 76 (44.4%) achieved overall NEDA (i.e., attainment and maintenance of both clinical and MRI NEDA) over 2 years (Fig. 2a). Among the 181 patients who had measurements over 2 years for EDSS worsening and relapse, 131 (72.4%) maintained clinical NEDA; among the 161 patients who had data over 2 years for Gd + lesions and new or newly enlarging T2 lesions, 99 (61.5%) maintained MRI NEDA.

**Fig. 3:**
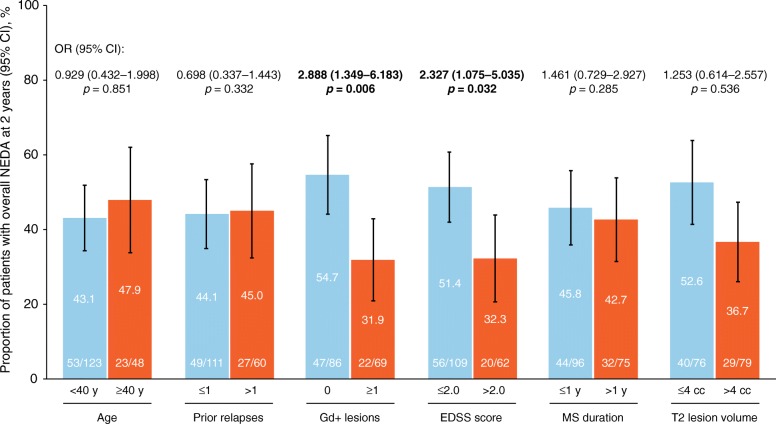
Overall NEDA over 2 years stratified by baseline characteristics. Patients who did not achieve NEDA at year 1 and who had missing data at year 2 were included in the analysis population. Statistically significant outcomes are shown in bold. CI = confidence interval; EDSS = Expanded Disability Status Scale; Gd + = gadolinium enhancing; MS = multiple sclerosis; NEDA = no evidence of disease activity; OR = odds ratio

A significantly higher proportion of patients with no baseline Gd + lesions (54.7%) than with ≥ 1 baseline Gd + lesion (31.9%) achieved overall NEDA over 2 years (*p* = 0.006) (Fig. 3). Similarly, patients with baseline EDSS scores ≤ 2.0 were significantly more likely to achieve overall NEDA than those with baseline EDSS scores > 2.0 (*p* = 0.032). Baseline differences in age, prior relapses, MS disease duration, and T2 lesion volume were not associated with significant differences in overall NEDA over 2 years (Fig. 3).

**Fig. 4:**
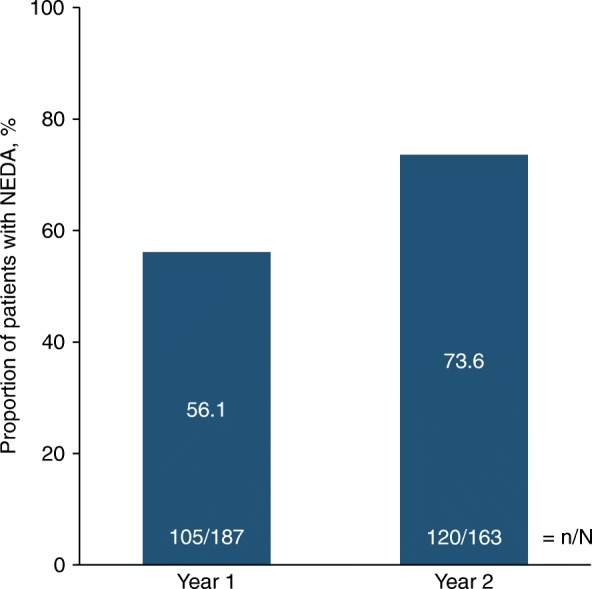
Proportion of patients with overall NEDA during the first or second year of natalizumab treatment. Only patients with no missing data at the time of the assessment were included. NEDA = no evidence of disease activity

In a yearly analysis of overall NEDA, 105 of 187 patients (56.1%) had NEDA from baseline to 1 year of natalizumab treatment and 120 of 163 (73.6%) exhibited NEDA from 1 year to 2 years (Fig. 4).

### Clinical and MRI disease activity

**Fig. 5:**
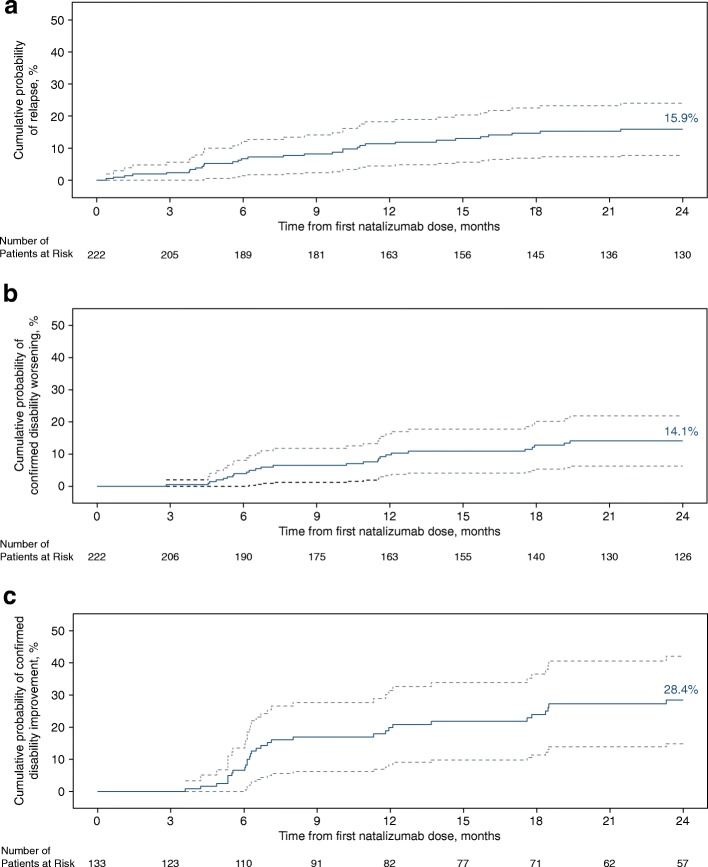
Cumulative probability of (**a**) relapse, (**b**) 24-week–confirmed disability worsening, and (**c**) 24-week–confirmed disability improvement over 2 years. Cumulative probabilities are based on Kaplan-Meier analysis or the Cox proportional-hazards model. Solid line shows the estimated cumulative probability; dashed lines show the 95% CI. Relapses in the year prior to starting natalizumab were reported by the patient. On-treatment relapses were reported by the physician. For disability outcomes, time point listed is for onset of EDSS increase or decrease, which was then confirmed 24 weeks later. CI = confidence interval; EDSS = Expanded Disability Status Scale

On individual measures of disease activity, 153 of 184 patients (83.2%) experienced no relapses and 148 of 174 (85.1%) experienced no confirmed disability worsening (Fig. 2b). In 100 of 160 patients (62.5%), no new or enlarging T2 lesions were observed, and most patients (149 of 160; 93.1%) had no new Gd + lesions. Patients had a significantly lower ARR after receiving natalizumab for 2 years (0.146) than during the year prior to treatment initiation (1.437; *p <* 0.001). Over 2 years of treatment, the cumulative probability of relapse was 15.9% (Fig. 5a). At 2 years, the cumulative probability of 24-week–confirmed disability worsening was 14.1% (Fig. 5b), and the cumulative probability of 24-week–confirmed improvement was 28.4% (Fig. 5c).

The mean (standard deviation) number of new or enlarging T2 lesions was 0.98 (2.72) after 1 year on natalizumab and 0.45 (1.83) after 2 years (Table 2). The mean number of Gd + lesions significantly decreased from baseline after 1 and 2 years on natalizumab (*p* < 0.001 for both).

**Table 2:** Summary of MRI measures

MRI measure	1 year	2 years
No. of new/newly enlarging T2 lesions	(*n* = 186)	(*n* = 157)
Mean (SD)	0.98 (2.72)	0.45 (1.83)
Median (min, max)	0 (0, 23)	0 (0, 11)
No. of Gd + lesions	(*n* = 191)	(*n* = 158)
Mean (SD)	0.03 (0.24)	0.07 (0.39)
Median (min, max)	0 (0, 3)	0 (0, 3)
Mean change from baseline (95% CI)	−2.78 (−3.98, −1.58)	−2.78 (−4.18, − 1.37)
*p*-value	**< 0.001**	**< 0.001**

*CI* confidence interval, *Gd +* gadolinium enhancing, *MRI* magnetic resonance imaging, *SD* standard deviation

*p*-values are for change from baseline and are based on a Wilcoxon signed-rank test. Statistically significant *p-*values are shown in bold. *p*-values were not calculated for new/newly enlarging T2 lesions

### Cognition, HRQoL, and work capacity

**Fig. 6:**
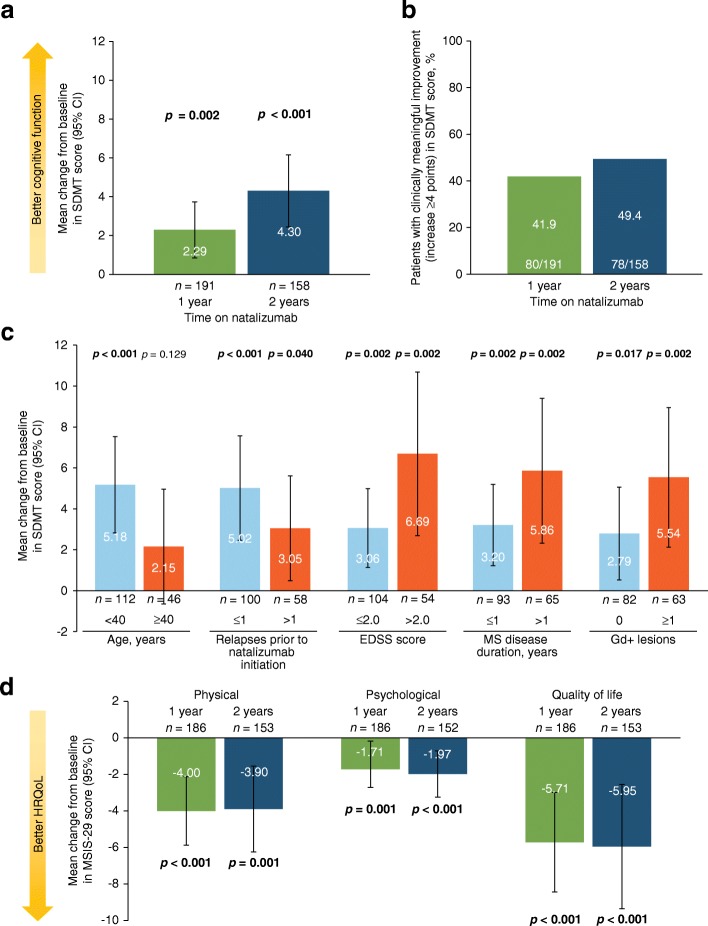
**a** Change from baseline to 1 and 2 years in SDMT score, **b** percentage of patients with clinically significant improvement in SDMT score (defined as an increase ≥4 points) at 1 and 2 years, **c** change in SDMT score from baseline to 2 years by baseline characteristics, and **d** change from baseline to 1 and 2 years in MSIS-29 physical, psychological, and quality-of-life scores. *p*-values are based on a paired *t*-test. CI = confidence interval; MSIS-29 = Multiple Sclerosis Impact Scale; SDMT = Symbol Digits Modality Test

Cognitive function, as measured by change from baseline in SDMT score, improved significantly after 1 and 2 years on natalizumab (Fig. 6a). A clinically significant cognitive improvement (i.e., an increase in SDMT score of ≥ 4 points [22]) was observed in 41.9% of patients at 1 year and 49.4% of patients at 2 years (Fig. 6b). In a subgroup analysis of mean change from baseline to 2 years, patients exhibited statistically significant improvement in SDMT score regardless of the number of relapses prior to natalizumab initiation, baseline EDSS score, MS disease duration at baseline, or baseline number of Gd + lesions (Fig. 6c).

Like SDMT scores, patient HRQoL, as measured by change from baseline in MSIS-29 physical, psychological, and quality-of-life scores, improved significantly after 1 and 2 years on natalizumab (Fig. 6d). Capacity for work as measured by WPAI also generally improved after 1 and 2 years of treatment (Table 3). At 2 years, patients showed significant improvements from baseline in hours missed from work per week due to MS (2.84 fewer hours missed than at baseline; *p* = 0.024) and regular activities affected by MS (*p* = 0.011).

**Table 3:** Summary of capacity for work as measured by WPAI at 1 and 2 years

WPAI assessment	Baseline	1 year			2 years		
	Score, mean (SD)	Score, mean (SD)	Change from baseline, mean (95% CI)	*p*-value	Score, mean (SD)	Change from baseline, mean (95% CI)	*p*-value
Hours missed from work per week because of MS	5.4 (12.73)	1.9 (6.10)	−1.69 (−3.57, 0.18)	0.076	1.5 (4.66)	−2.84 (−5.29, − 0.38)	**0.024**
	(*n* = 144)	(*n* = 126)	(*n* = 108)		(*n* = 109)	(*n* = 87)	
Hours missed from work per week for non-MS reasons	2.9 (5.61)	3.3 (7.09)	0.57 (−1.09, 2.22)	0.498	2.9 (5.80)	−0.73 (−2.35, 0.89)	0.373
	(*n* = 147)	(*n* = 126)	(*n* = 112)		(*n* = 109)	(*n* = 92)	
Hours worked per week	33.1 (16.30)	31.9 (15.10)	−1.53 (−4.68, 1.61)	0.336	33.9 (14.64)	2.38 (−1.00, 5.76)	0.166
	(*n* = 146)	(*n* = 124)	(*n* = 108)		(*n* = 112)	(*n* = 94)	
Work productivity affected by MS^a^	2.6 (2.62)	2.2 (2.52)	−0.15 (−0.62, 0.31)	0.515	2.3 (2.54)	−0.21 (− 0.83, 0.41)	0.500
	(*n* = 144)	(*n* = 125)	(*n* = 110)		(*n* = 111)	(*n* = 95)	
Regular activities affected by MS^b^	3.7 (2.96)	3.3 (3.05)	−0.37 (− 0.79, 0.04)	0.078	3.0 (2.98)	−0.61 (−1.08, − 0.14)	**0.011**
	(*n* = 218)	(*n* = 187)	(*n* = 185)		(*n* = 150)	(*n* = 148)	

*CI* confidence interval, *MS* multiple sclerosis, *SD* standard deviation, *WPAI* Work Productivity and Activity Impairment Questionnaire

*p*-values are for change from baseline and are based on a paired *t*-test. Statistically significant *p-*values are shown in bold

^a^Patients were asked “During the past 7 days, how much did your problem affect your productivity while you were working?” Patients provided an answer from 0 to 10, with 0 indicating that the problem had no effect on their work and 10 indicating that the problem completely prevented them from working

^b^Patients were asked “During the past 7 days, how much did your problem affect your ability to do your regular daily activities, other than work at a job?” Patients provided an answer from 0 to 10, with 0 indicating that the problem had no effect on their daily activities and 10 indicating that the problem completely prevented them from doing their daily activities

### OCT and Vision

In the subgroup of patients with OCT assessments at baseline and 2 years (*n* = 50), a mild decrease in RNFL thickness as measured by OCT was observed. Overall mean change in RNFL thickness from baseline for this subgroup at 2 years was − 1.365 μm (95% CI: − 2.326 μm, − 0.404 μm; *p* = 0.005). The percentage change from baseline was − 1.377 (95% CI: − 2.366, − 0.387; *p* = 0.006).

**Fig. 7:**
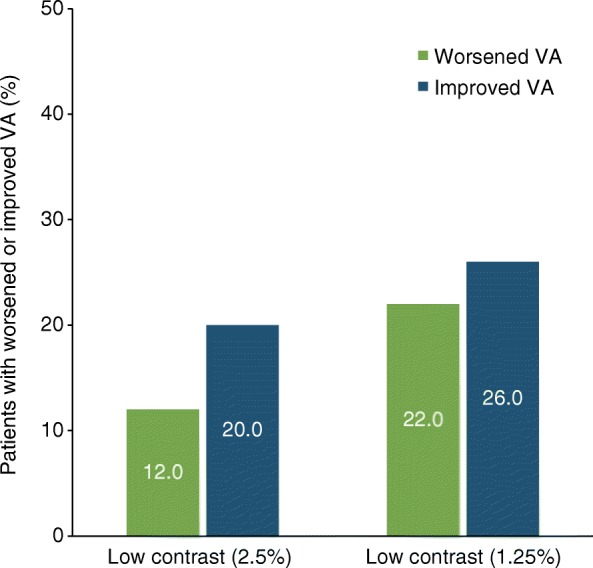
Proportions of patients with worsened VA (defined as a loss of ≥7 letters) or improved VA (defined as an increase of ≥7 letters) in both eyes over 2 years in the STRIVE OCT subgroup per protocol population (*n* = 50)

Over 2 years, worsening in VA at high contrast (100%), defined as a loss of ≥5 letters [20], was experienced by 5 of 46 patients (10.9%). Worsening in VA at low contrast (2.5 and 1.25%), defined as a loss of ≥7 letters [20], was experienced by 6 of 50 patients (12.0%) and 11 of 50 patients (22.0%), respectively (Fig. 7). Improvement in VA at high contrast, defined as an increase of ≥5 letters [21], was experienced by 11 of 46 patients (23.9%) over 2 years. At low contrast (2.5 and 1.25%), improvement in VA, defined as an increase of ≥ 7 letters [21], was experienced by 10 of 50 patients (20.0%) and 13 of 50 patients (26.0%), respectively (Fig. 7).

### Safety

Of the 222 patients in the ITT population, 13 (5.9%) experienced ≥ 1 treatment-emergent SAE during the first 2 years of STRIVE (Table 4). One death was recorded; the cause of death was listed as “subdural haematoma,” as a result of traumatic brain injury, and the event was judged by the investigator to be unrelated to treatment. No PML cases occurred in the study.

**Table 4:** SAEs during 2 years of natalizumab treatment^a^

*n* (%)	Natalizumab-treated patients (*N* = 222)
Patients with ≥1 SAE	13 (5.9)
Patients with ≥1 treatment-related SAE	1 (0.5)^b^
Patients with ≥1 SAE leading to death	1 (0.5)^c^
SAE by preferred term^d^	
Suicide attempt	2 (0.9)
Acute kidney injury	1 (0.5)
Anaphylactic reaction^e^	1 (0.5)
Bronchial obstruction	1 (0.5)
Clostridium difficile colitis	1 (0.5)
Conversion disorder^f^	1 (0.5)
Hydronephrosis	1 (0.5)
Hyperkalemia	1 (0.5)
Hypotension	1 (0.5)
Ileus	1 (0.5)
Melanoma recurrent	1 (0.5)
Migraine	1 (0.5)
Overdose	1 (0.5)
Posttraumatic headache	1 (0.5)
Radial nerve palsy	1 (0.5)
Respiratory failure	1 (0.5)
Unresponsive to stimuli	1 (0.5)
Ureterolithiasis	1 (0.5)

*SAE* serious adverse event

^a^SAEs with an onset date within 2 years of the first natalizumab dose are included

^b^Anaphylactic reaction

^c^The cause of death was subdural hematoma and was unrelated to study treatment. The subject received only 1 dose of natalizumab. The death was reported > 3 months after the subject received natalizumab

^d^SAE occurring while on natalizumab treatment or within 30 days of the last dose are reported. In addition to the terms listed, MS relapse was reported as an SAE for 4 patients (1.4%)

^e^No diagnostic test was performed to evaluate this event. The patient was transported to the emergency department and observed < 4 h but was not hospitalized for evaluation of the event. The patient was discharged and has not had an anaphylactic reaction since then

^f^The patient was hospitalized for > 24 h

## Discussion

The primary objective of the STRIVE 2-year interim analysis was to determine the effectiveness of natalizumab at establishing and maintaining NEDA in patients with early RRMS. In addition to traditional measures of MS treatment outcome, NEDA has increasingly been used as a treatment goal [23, 24]. In this analysis, 44.4% of patients initiating natalizumab treatment early in their disease course (< 3 years from diagnosis) attained overall NEDA based on clinical and neuroimaging criteria. In a yearly analysis, a higher percentage of patients attained NEDA in the second year on natalizumab treatment than in the first (73.6% vs. 56.1%), which is consistent with reports of increasing natalizumab effectiveness with longer exposure [2]. The proportions of patients who achieved overall, clinical, and MRI NEDA over 2 years raises the possibility that a significant number of patients might have experienced relapse in the absence of observable disease activity on MRI. In this regard, it may be relevant to note that while MRI follow-ups in this study were limited to white matter lesions, a recent longitudinal study identified a correlation between natalizumab-associated reduction in relapses and reduction in new cortical (grey matter) lesions but not white matter lesions [25].

It has been suggested that commonly used definitions of NEDA, such as a complete absence of new T2 lesions, might represent too stringent a clinical practice goal [26]. More specifically, some studies have suggested that risk of EDSS progression in RRMS patients treated with interferon beta is significantly associated only with substantial MRI activity (i.e., ≥ 3 new T2 lesions) [27]. Therefore, the NEDA criteria used in this study may have yielded a conservative estimate of the proportion of patients who responded favorably to natalizumab treatment.

Significantly more patients without than with baseline Gd + lesions on MRI achieved overall NEDA. Having minimal or no disability when initiating treatment was associated with achieving clinical NEDA after 2 years of treatment. Long-term follow-up will determine if the difference between these subgroups in achieving NEDA persists for > 2 years.

At 2 years, the cumulative probability of 24-week–confirmed EDSS improvement (28.4%) appeared higher than the cumulative probability of 24-week–confirmed EDSS worsening (14.1%), and 196 of 222 patients (88.2%) had no 24-week–confirmed EDSS worsening. Both disability worsening and disability improvement appeared to be most common during the first year of treatment, though disability improvement continued to increase during the second year. These results are consistent with an AFFIRM post hoc analysis that also demonstrated that natalizumab increased the cumulative probability of disability improvement in MS patients compared with placebo over a 2-year period [28]. However, it should be noted that the AFFIRM patients had longer disease durations than the patients in this analysis.

Studies of the natural history of cognitive impairment measured by SDMT in MS have demonstrated increasing cognitive impairment (reflected in decreasing SDMT scores) over time in the absence of DMTs [29–31]. In STRIVE, SDMT scores improved significantly from baseline after 1 year of treatment, an effect sustained through the second year of treatment. After 2 years on natalizumab, nearly half (49.4%) of treated patients exhibited clinically significant cognitive improvement. While practice effects have been reported for the SDMT test administered at monthly intervals [32], in this study, cognitive performance was assessed annually, making confounding by a practice effect less likely. Quality of life (measured by MSIS-29) also significantly improved in patients following 1 and 2 years of treatment. Like STRIVE, other studies in RRMS patients have shown natalizumab-associated improvements in both SDMT and MSIS-29 scores over approximately 12–24 months [33, 34], supporting the benefits of natalizumab on the quality-of-life and cognitive outcomes observed here.

As STRIVE is an open-label, single-arm study, it has several limitations, the most significant of which is the lack of an internal comparator group. Therefore, placebo effects on the clinical outcomes cannot formally be ruled out. However, our results on cognitive performance and EDSS associated with natalizumab are consistent with those reported in the AFFIRM placebo-controlled trial [35]. The open-label nature of STRIVE could introduce selection bias in the study population; however, this study was open to all subjects with early MS and permitted even subjects with very active disease to enroll. Furthermore, since STRIVE was initiated prior to the evidence that anti-JCV antibody levels (“index”) may differentiate PML risk in natalizumab-treated MS patients without prior immunosuppressant use [11, 36], anti-JCV antibody index was not assessed in this study.

Results from the OCT substudy indicate that over 2 years, natalizumab-treated patients demonstrated relative preservation of RNFL thickness in comparison with analyses of other MS cohorts [20, 37] and with non-MS subjects [12]. Patients also had relatively mild losses of RNFL thickness and low-contrast letter acuity over 2 years. A numerically higher proportion of patients has improved VA than worsened VA; rates of improvement in VA were consistent with those reported for natalizumab-treated patients in the AFFIRM study [21]. Furthermore, rates of worsened VA among natalizumab-treated patients appeared similar to those in a non-MS sample [12].

Recently, a study in a real-world data set used propensity-score matching to demonstrate that among first-line therapies, natalizumab can provide greater reduction of MS disease activity than interferons or glatiramer acetate [38]. In addition, a decision analysis approach simulating a head-to-head comparison of the risks and benefits of glatiramer acetate, fingolimod, and natalizumab as first-line treatment for MS demonstrated a clear advantage for natalizumab [39]. Further studies assessing natalizumab use in early RRMS are crucial to inform the benefit/risk profile of natalizumab under real-world treatment scenarios.

## Conclusions

These 2-year results support the effectiveness of natalizumab in treating anti-JCV antibody negative patients with early RRMS. Over 2 years, nearly half of early RRMS patients achieved NEDA. During the second year, nearly 75% of patients exhibited NEDA. Patients also experienced significant cognitive and quality-of-life benefits over 2 years on natalizumab.

## Additional file

Additional file 1: Ethics committees that approved the STRIVE study. (DOCX 13 kb)

## Data Availability

The datasets generated and/or analyzed during this study are not publicly available. Requests for deidentified data should be made to Biogen via established company data-sharing policies and processes as detailed on the website http://clinicalresearch.biogen.com/.

## Abbreviations

ARR: Annualized relapse rate
CI: Confidence interval
DMT: Disease-modifying therapy
EDSS: Expanded Disability Status Scale
ELISA: Enzyme-linked immunosorbent assay
Gd +: Gadolinium enhancing
HRQoL: Health-related quality of life
ITT: Intent to treat
JCV: JC virus
MRI: Magnetic resonance imaging
MS: Multiple sclerosis
MSIS-29: Multiple Sclerosis Impact Score
NEDA: No evidence of disease activity
OCT: Optical coherence tomography
PML: Progressive multifocal leukoencephalopathy
PRO: Patient-reported outcome
RNFL: Retinal nerve fiber layer
RRMS: Relapsing-remitting multiple sclerosis
SDMT: Symbol Digit Modalities Test
SAE: Serious adverse event
STRIVE: Observational Study of Tysabri in Early Relapsing-Remitting MS in Anti-JCV Antibody Negative Patients
TOP: Tysabri Observational Program
VA: visual acuity
WPAI: Work Productivity and Activity Impairment Questionnaire
