# Supplementary material for: Outcomes of natalizumab treatment within 3 years of relapsing-remitting multiple sclerosis diagnosis: a prespecified 2-year interim analysis of STRIVE
Source: BMC Neurol. 2019 Jun 8;19:116. doi: 10.1186/s12883-019-1337-z (PMC6555913; doi:10.1186/s12883-019-1337-z)
Supplement: Supplementary file 1 — Ethics committees that approved the STRIVE study. (DOCX 13 kb) [file 12883_2019_1337_MOESM1_ESM.docx]

| **Name of Institutional Review Board (IRB)/**  **Ethics Committee (EC)** | **Location** |
| --- | --- |
| Cleveland Clinic IRB | Cleveland, OH |
| University of Maryland School of Medicine Human Research Protections Office | Baltimore, MD |
| UC Davis IRB | Davis, CA |
| Copernicus Group Independent Review Board | Durham, NC |
| Peoria IRB | Peoria, IL |
| Lahey Clinic IRB | Burlington, MA |
| Committee for the Protection of Human Subject in Research | Worcester, MA |
| Michigan State University Biomedical and Health IRB | Lansing, MI |
| Georgetown University Hospital IRB | Washington, DC |
| Western Institutional Review Board | Puyallup, WA |
| University of Kentucky IRB | Lexington, KY |
| Providence Health & Services IRB | Spokane, WA |
| St. Vincent Institutional Review Board | Indianapolis, IN |
| Altru Health System IRB | Grand Forks, ND |
| Penn State Milton S. Hershey Medical Center IRB | Hershey, PA |
| Health Sciences Campus | Los Angeles, CA |
| Quorum Review IRB | Seattle, WA |
| Scripps IRB | Claremont, CA |
| Ochsner Institutional Review Board | New Orleans,   LA |
| SUNY Stony Brook’s IRB | Stony Brook, NY |
| Tufts Medical Center IRB | Boston, MA |
| Weill Cornell Medical College IRB | New York, NY |
| Johns Hopkins University IRB | Baltimore, MD |
| Shepherd Research Review committee | Shepherdstown, WV |
| Alta Bates Summit Investigational Review Board | Berkeley, CA |
| University of Texas Southwestern Investigational Review Board | Dallas, TX |
| Centrastate Investigational Review Board | Freehold, NJ |
| University Hospitals Case Medical Center | Cleveland, OH |
| Christiana Care Institutional Review Board | Newark, DE |
| Abington Memorial Hospital IRB | Abington, PA |
| Institutional Review Board of St. Elizabeth’s Medical Center | Brighton, MA |
| NYU School of Medicine IRB | New York, NY |
| Rush University Medical Center IRB | Chicago, IL |
| Beth Israel Deaconess Medical Center IRB | Boston, MA |
| Northwestern University IRB | Evanston, IL |
